# Characteristics of endoplasmic reticulum stress in colorectal cancer for predicting prognosis and developing treatment options

**DOI:** 10.1002/cam4.5874

**Published:** 2023-03-31

**Authors:** Jingbo Geng, Yunkai Guo, Mingjie Xie, Zhipeng Li, Peng Wang, Donghui Zhu, Jing Li, Xiaopeng Cui

**Affiliations:** ^1^ Department of General Surgery Affiliated Hospital of Nantong University Nantong 226001 China; ^2^ Medical School of Nantong University Nantong 226001 China; ^3^ Cancer Research Center Nantong Tumor Hospital & Affiliated Tumor Hospital of Nantong University Nantong 226001 China

**Keywords:** chemotherapy resistance, colorectal cancer, endoplasmic reticulum stress, immunotherapy, prognosis

## Abstract

**Background:**

An increasing body of evidence supports an essential role for endoplasmic reticulum stress (ERS) in colorectal cancer (CRC). In this study, we developed an ERS‐related genes (ERSRGs) model to aid in the prognostic evaluation and treatment of CRC patients.

**Methods:**

The training set and validation set data were extracted from The Cancer Genome Atlas (TCGA) and Gene Expression Omnibus (GEO), respectively. ERSRGs were obtained from the GeneCards database. A prognostic risk scoring model was constructed using the least absolute shrinkage and selection operator (LASSO) along with univariate Cox regression analysis. To further predict the probability of survival for patients at 1, 2, and 3 years, a nomogram was devised. The advantages of the prognostic risk score model in screening patients’ sensitive to chemotherapy and immunotherapy were analyzed by drug sensitivity analysis and immune correlation analysis. Finally, hub genes associated with poor prognosis in the risk model were screened by Protein–protein interaction (PPI) network and their expression was validated using clinical specimens.

**Results:**

A risk model for overall survival (OS) was developed using 16 ERSRGs associated with prognosis. Through analyses, we demonstrated a high degree of reliability for the prognostic risk scoring model. The constructed nomograms performed well in predicting patient survival over 1, 3, and 5 years. The calibration curve and decision curve analysis (DCA) supported a high degree of accuracy for the model. Patients in the low‐risk group had a lower IC50 for the common chemotherapy drug, 5‐FU, and responded better to immunotherapy. hub poor prognostic genes were validated in CRC clinical specimens.

**Conclusion:**

We have identified and validated a new ERS prognostic marker that can accurately predict the survival status of CRC patients for clinicians and better provide personalized treatment plans.

## Graphical abstract

This study found that the endoplasmic reticulum stress risk score model can accurately predict the prognosis of CRC patients. The endoplasmic reticulum stress risk score model can also provide individualized plans for patient treatment. We also provided clinical samples to validate the expression of prognostic key genes in the model.

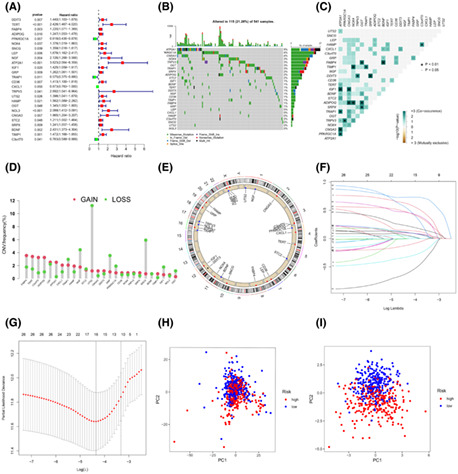

## 1 INTRODUCTION

Colorectal cancer (CRC) is a highly prevalent malignancy, causing a large number of deaths each year.^1^, ^2^ There are many causes of CRC, such as poor diet, unhealthy living habits, and environmental and genetic factors.^3^ Although there are new options in the treatment strategy for CRC. its mortality continues to rise in most countries and in developing countries, in particular.^4^ Currently, the treatment of CRC includes surgical resection, chemotherapy, immunotherapy and radiotherapy. Although such treatments have improved the survival rate of patients to a certain degree, their prognosis is still less than ideal, with approximately 30%–50% of patients experiencing recurrence and progression of the disease after these conventional treatments.^5^, ^6^, ^7^ Thus, a more in‐depth study of the reasons behind the development and poor outcome of CRC is necessary to more effectively guide clinical treatment. In this study, we sought to explore the functional role and clinical prognostic value of endoplasmic reticulum stress (ERS) in CRC.

When the endoplasmic reticulum (ER) receives stress signals, the homeostasis of the ER is disrupted, which affects a variety of cellular processes, including cell growth, differentiation, and induction of apoptosis.^8^ In different types of tumors, various oncogenic factors and transcriptional and metabolic abnormalities act synergistically to bring tumor cells into a sustained state of ERS. Activation of ERS and its downstream signaling pathways coordinate a dynamic balance of proteins that becomes a key factor in tumor growth, angiogenesis, resistance to radiotherapy, and response to immunotherapy.^9^, ^10^, ^11^ When misfolded or unfolded proteins exceed the capacity of ER processing, it leads to ERS and stimulates unfolded protein response (UPR). UPR is mainly composed of three key pathways: IRE1α, PERK, and ATF6, which jointly regulate the stress response of cells.^11^ Previous studies have found that in breast cancer cell model, ATF6, as one of the three main pathways of UPR, is activated and regulated by mutant p53 during ERS, which promotes tumor cell invasion, metastasis, and chemotherapy resistance.^12^ Another study showed that the ERS effector PERK is upregulated in drug‐resistant cells of CRC and leads to tumor cell invasion and chemoresistance via the PERK/Nrf2/MRP1 axis.^13^ When ERS occurs in immune cells, it also alters their normal functional state, thereby affecting the development of malignancy.^11^ Studies have found that ERS can cause the immune escape of breast cancer cells by regulating specific miRNAs, leading macrophages to up‐regulate PD‐L1.^14^ However, the effect of ERS on CRC remains unclear.

In this study, we envisioned that ERS affects CRC development as well as leads to CRC chemoresistance through complex molecular mechanisms. Therefore, we analyzed the expression and correlation of ERS‐related genes (ERSRGs) in CRC and constructed a prognostic risk score model. Our prognostic model performed well in predicting patients' prognosis conveniently and was able to differentiate between patients’ sensitive to chemotherapy and immunotherapy. In addition, by combining clinically relevant information, we also constructed nomograms. Taken together, we provide a new approach for the stratification of prognostic risk and navigation of clinical treatment options for CRC.

## 2 METHODS

### 2.1 Data and clinical information access

Genetic and clinically relevant information in the training set was extracted from The Cancer Genome Atlas (TCGA) established by American Cancer Center and no data has been eliminated, including 568 CRC and 44 normal specimens. All data for the validation set are from the GSE40967 and GSE17538 datasets in the Gene Expression Omnibus (GEO). Finally, 856 ERSRGs were screened from the online website GeneCards.

### 2.2 Functional enrichment analysis of ERSRGs in normal and tumor samples

Analysis of differentially expressed ERSRGs samples was done using the “limma”, “pheatmap”, and “BiocManager” packages in R software. (|log2FC| = 0.585; FDR <0.05). Gene ontology (GO) and Kyoto Encyclopedia of Genes and Genomes (KEGG) enrichment analysis of differentially expressed genes (DEGs) was conducted by R software.

### 2.3 Development and validation of risk scoring models

The training and validation set of this model are using CRC samples from the TCGA sample and the GSE40967 cohort, respectively. Screening for prognosis‐related genes from DEGs was done with univariate Cox regression analysis (*p* < 0.05). Visualization of mutations and associations of prognosis‐related genes in CRC samples by “maftools” in R software. Copy number variant (CNV) information for CRC was obtained from the UCSC Xena database. CNV frequencies were then calculated for prognosis‐related genes and the results were visualized via bivariate histograms and circle plots. The best prognostic risk score model was established using “glmnet” and “survivor” in R software. The calculation formula is as follows: risk score $=\sum_{1}^{i}\left(\text{Coefi}*\text{ExpGenei}\right)$, where “Coef” and “ExpGene” were generated by least absolute shrinkage and selection operator (LASSO) Cox regression analysis. The Kaplan–Meier plot and log‐rank test combined with LASSO Cox regression were used to evaluate survival differences between the high‐ and low‐risk groups. The time‐dependent ROC curve was plotted using “survivalROC” in the R software, and the predictive accuracy of the model was assessed by the area under the curve (AUC). Finally, validation is performed in GSE40967.

### 2.4 Construction and verification of nomograms

A nomogram integrating risk scores and other survival‐related factors was created using “rms,” “regplot,” and “survival” in the R software for prognostic assessment. AUC and correction curves were used to assess the predictive power. Decision curve analysis (DCA) was used to assess the clinical utility of the model.

### 2.5 Drug sensitivity and immune cell analysis

The “pRRophetic” in the R software was applied to predict the IC50 of different drugs in each sample. Immunoinfiltration analysis was performed using “CIBERSORT” in the R software. The tumor cellularity was calculated by the “estimation” and ESTIMATE algorithms in the R software. Microenvironment scores were calculated for each sample and then differences in stroma and immune cells were predicted based on risk model files.

### 2.6 Protein–protein interaction network analysis

DEGs were obtained from the RNA‐seq data of genes and risk files by “limma” in the R software (logFCfilter = 1; *p* < 0.05). GO and KEGG enrichment analysis of DEGs was performed using the “clusterProfiler” in R software to identify gene key functions. The protein–protein interaction (PPI) network of DEGs is calculated by website STRING (version: 11.0; https://string‐db.org/). Import the PPI network data into Cytoscape software (version: 3.9.1), visualize it, and obtain the hub gene through a plug‐in. Finally, the pivotal genes associated with poor prognosis were obtained using “limma,” “survivor,” and “survminer” in the R software.

### 2.7 Quantitative real‐time PCR

In this study, tissue samples were collected for real‐time quantitative PCR from 11 CRC patients treated at Nantong University Hospital.

RNA was isolated from fresh tissues using RNA extraction reagent (Invitrogen). Next, reverse transcription was performed. Finally, quantitative real‐time polymerase chain reaction (qRT‐PCR) was performed using SYBR‐Green mixture (Vazyme) and gene‐specific primers (Sangon Biotech). Glyceraldehyde‐3‐phosphate dehydrogenase was used as a standard reference (Table S1). Relative quantification of genes was calculated using the 2^−ΔΔCt^method.

### 2.8 Statistical analysis

Statistical analyses of the data in this study were performed using R version 4.2.0 software (*p* < 0.05). ThWilcoxon rank‐sum test was used to compare the difference between the high‐risk and low‐risk groups. The Kruskal–Wallis test was used to compare more than two groups. Cox regression analysis was performed to determine the predictive factors affecting overall survival (OS). OS is the time from grouping to death due to any reason. For subjects who have been lost before death, the last follow‐up time is usually calculated as the time of death.

## 3 RESULTS

### 3.1 Enrichment analysis of ERSRGs

First, we searched the GeneCard database for the keywords “endoplasmic reticulum stress” to identify a set of ERSRGs. Gene expression levels were compared using |log2FC| = 0.585 and FDR <0.05 as thresholds, and a total of 220 DEGs were detected (Figure S1A,B). The enrichment of DEGs was then analyzed, and GO and KEGG results showed that it was mainly associated with the ER, the endoplasmic reticulum lumen, ERS, ER protein processing, immune‐related signaling pathways, and CRC (Figure S1C,D). These results suggest that ERS plays a critical role in the development of CRC.

### 3.2 Screening EERSRGs and constructing risk score models

**FIGURE 1:**
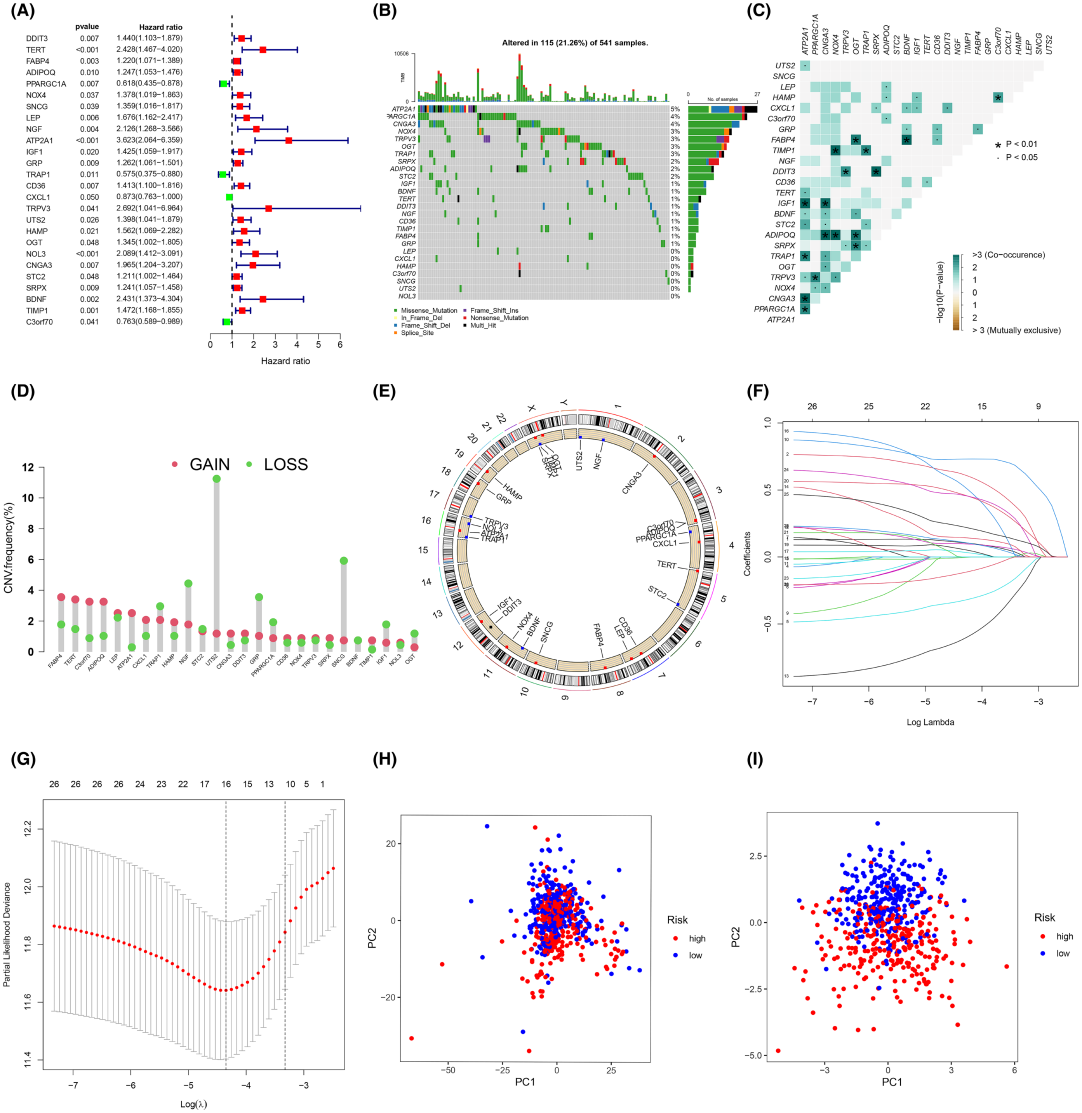
Construction of an ERS risk score model. (A) Sixteen ERSRGs associated with prognosis in patients with CRC. (B) Mutation frequencies in 16 ERSRGs in the training set. (C) Mutation co‐occurrence and exclusion analysis of 16 ERSRGs. (D) Frequency of CNV variants in 16 ERSRGs. (E) The circos plot depicts the location and expression levels of 16 ERSRGs on the chromosome. (F) LASSO coefficients of the 16 ERSRGs. (G) Selection of genes by LASSO Cox regression. (H) Principal component analysis based on all ERSRGs in CRC. (I) Principal component analysis based on ERS risk scores, distinguishing between tumor and normal samples in the training set. Red represents high‐risk patients and blue represents low‐risk patients.

Univariate Cox regression analysis of 220 DEGs identified 26 genes that were associated with prognosis (*p* < 0.05) (Figure 1A). First, the somatic mutations of ERSRGs in the sample were summarized. A total of 115 ERSRGs were mutated in 541 CRC samples with mutation information, with a frequency of 21.26% (Figure 1B). The mutation rates of ATP2A1, PARGC1A, and CNGA3 were ≥4%, with the mutation rate of ATP2A1 being the highest at 5%. However, LEP, CXCL1, HAMP, C3orf70, SNCG, UTS2, and NOL3 were not significantly mutated in CRC samples. Importantly, there was a significant signature of co‐existing mutations among the 26 ERSRGs associated with prognosis (Figure 1C). In addition, we analyzed the copy number changes of these ERSRGs and observed that the frequency of copy number alterations in these genes varied significantly, with FABP4, TERT, C3orf70, and ADIPOQ showing elevated copy numbers and NGF, UTS2, GRP, and SNCG showing copy number loss, together indicating abnormal expression of ERSRGs (Figure 1D,E). Given the prominent role of ERS in CRC, we sought to develop an ERS prognostic risk score model to more accurately assess the status of CRC patients. The clinical information of the CRC samples involved in the construction of the risk scoring model is shown in Table 1. Then 16 genes (DDIT3, TERT, PPARGC1A, ATP2A1, GRP, TRAP1, CD36, CXCL1, TRPV3, UTS2, OGT, NOL3, STC2, BDNF, TIMP1 and C3orf70) were screened by LASSO Cox regression analysis to construct the model (Figure 1F,G). The risk score was based on the following calculation: risk score = (0.107911822441034) × DDIT3 + (0.619078158487871) × TERT+(−0.326557849354418) × PPARGC1A+(0.731074292272508) × ATP2A1 + (0.0987929998696706) × GRP+(−0.616851679710394) × TRAP1 + (0.144576979652297) × CD36 + (−0.00434466921328452) × CXCL1 + (0.537783939824743) × TRPV3 + (0.0368714196385128) × UTS2 + (0.0579336086219892) × OGT+(0.467900143783939) × NOL3 + (0.110904469465992) × STC2 + (0.462790719366557) × BDNF+(0.226455155291319) × TIMP1 + (−0.11920477727086) × C3orf70(Table S2). The high‐risk group (*n* = 270) and low‐risk group (*n* = 270) in the training set were divided according to the median value of the risk score. The validation set was divided in the same way, and principal component analysis (PCA) showed that the model was effective in dividing the CRC sample into high‐risk and low‐risk groups (Figure 1H, I).

**TABLE 1:** Clinicopathological information of patients with CRC in two independent cohorts.

Characteristics	TCGA Total (*N* = 548)	GSE40967 Total (*N* = 584)
Age		
>65	330 (60.22%)	367 (62.84%)
≤65	218 (39.78%)	216 (36.99%)
Gender		
Female	256 (46.72%)	263 (45.03%)
Male	292 (53.28%)	321 (54.97%)
Status		
Alive	439 (80.11%)	384 (65.75%)
Dead	109 (19.89%)	194 (33.22%)
Unknown	0 (0%)	6 (1.03%)
Stage		
Stage 0	0 (0%)	3 (0.51%)
Stage I	96 (17.52%)	38 (6.51%)
Stage II	210 (38.32%)	271 (46.40%)
Stage III	149 (27.19%)	210 (35.96%)
Stage IV	78 (14.23%)	60 (10.27%)
Unknown	15 (2.74%)	2 (0.34%)
T		
T1	15 (2.74%)	12 (2.05%)
T2	96 (17.52%)	49 (8.39%)
T3	373 (68.07%)	379 (64.90%)
T4	63 (11.50%)	119 (20.38%)
Tis	1 (0.18%)	3 (0.51%)
Unknown	0 (0%)	22 (3.77%)
N		
N0	323 (58.94%)	313 (53.60%)
N1	130 (23.72%)	137 (23.46%)
N2	94 (17.15%)	100 (17.12%)
N3	0 (0%)	6 (1.03%)
NX	1 (0.18%)	6 (1.03%)
Unknown	0 (0%)	22 (3.77%)
M		
M0	408 (74.45%)	498 (85.27%)
M1	77 (14.05%)	61 (10.45%)
MX	55 (10.04%)	3 (0.51%)
Unknown	8 (1.46%)	22 (3.77%)

### 3.3 Risk prediction and survival status for the training and validation set samples

**FIGURE 2:**
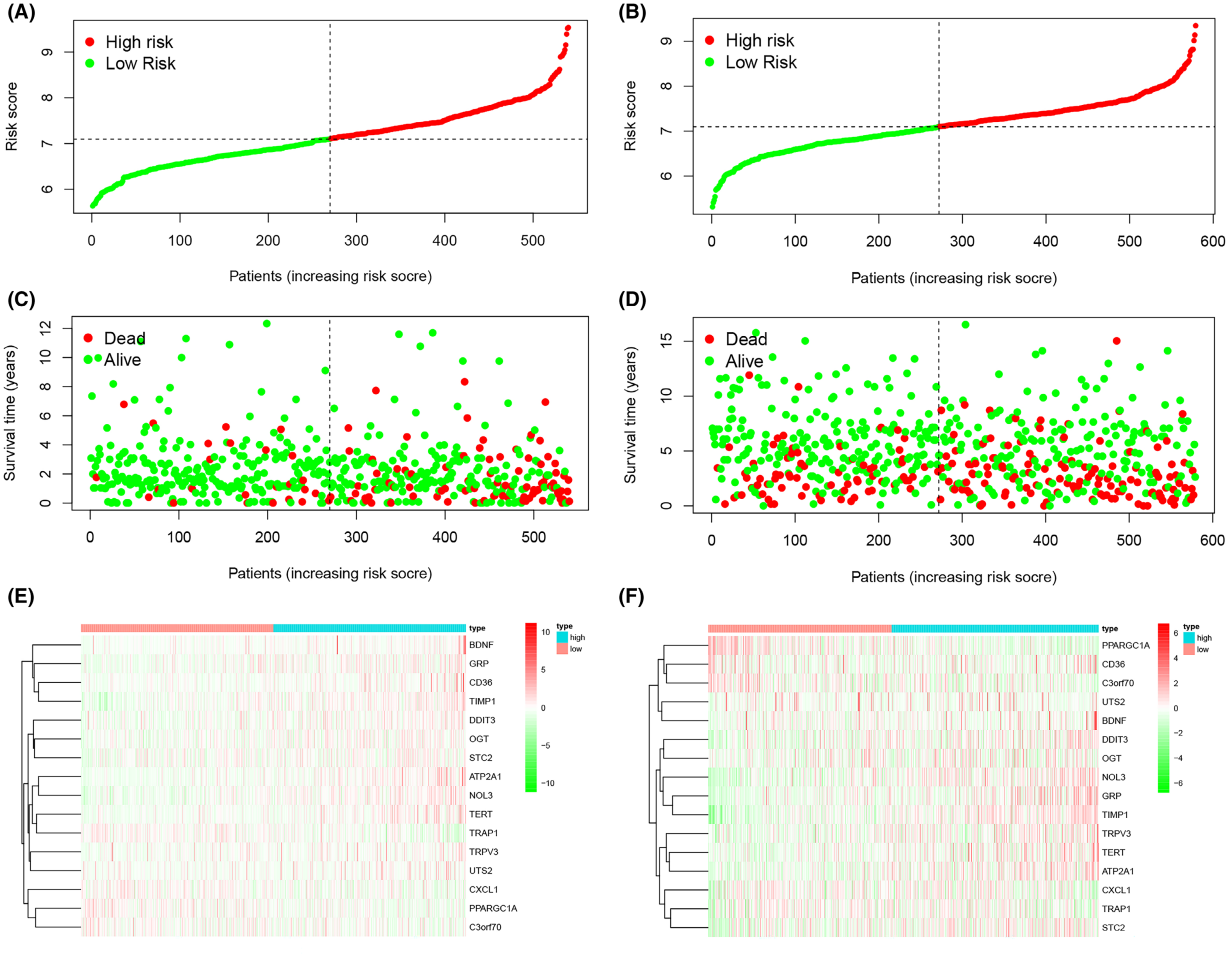
Risk prediction of patients in the ERS model and the expression levels of genes included in the model. (A, B) Patient scores in the training and validation sets. (C, D) Survival rates in the high‐ and low‐risk groups in the training and validation sets. (E, F) Heat map of gene expression levels of risk models in the training and validation sets.

Risk curves were generated from the samples in the dataset, where risk scores were visualized for both groups of patients (Figure 2A,B). The analysis found that the prognosis of patients in the high‐risk group was poor and worse as the risk score increased, along with an increase in mortality (Figure 2C,D). Next, the risk histogram visually assesses the survival status in the risk score model (Figure S2A,B). Patients with low‐risk scores were found to have significantly higher survival rates, further demonstrating that the model can accurately stratify patients. Moreover, we used thermography to compare the expression levels of ERSRGs identified as affecting patient prognosis between the two risk groups. BDNF, GRP, CD36, TIMP1, DDIT3, OGT, STC2, ATP2A1, NOL3, TERT, TPRV3 and UTS2 had high levels in the high‐risk group and low levels of PPARGC1A, C3orf70, CXCL1, and TARP1(Figure 2E,F). We also found a significant association between these genes (Figure S2C,D).

### 3.4 Predictive power of the model

**FIGURE 3:**
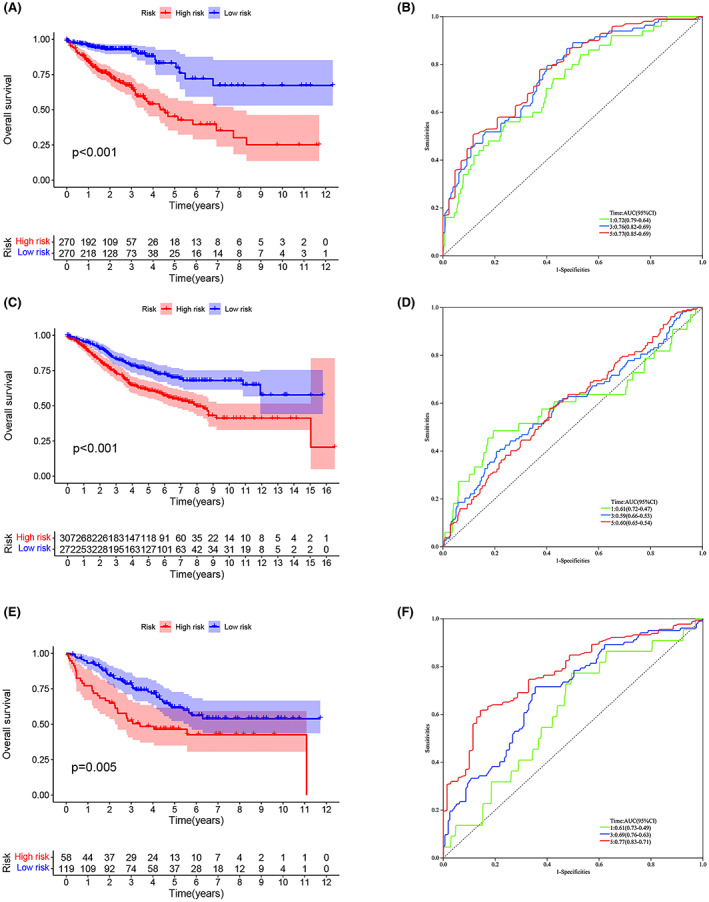
Relationship between the ERS prognostic risk model and clinicopathological features and predictive value for survival. (A) Kaplan–Meier survival curves for the training set. (B) ROC curves for the risk model in the training set. (C) Kaplan–Meier survival curves for the validation set (GSE40967). (D) ROC curves for the risk model in the validation set (GSE40967). (E) Kaplan–Meier survival curves for the validation set (GSE17538). (F) ROC curves for the risk model in the validation set (GSE17538).

In the training set, the OS of the high‐risk group was lower than that of the low‐risk group (*p* < 0.001) (Figure 3A). ROC curves were used to validate the predictive effect of the model on patient OS, with AUC results of 0.72, 0.76, and 0.77 at 1, 3, and 5 years, respectively (Figure 3B). To check the reliability of the model, tests were conducted on the validation set (GSE40967). Patients in the high‐risk group had a lower OS compared to the low‐risk group (*p* < 0.001) (Figure 3C). The AUC values of the validation set are shown in Figure 3D. In order to further verify the accuracy of the prognostic model, we verified it again in the GSE17538 dataset (*p* < 0.005). The results showed that the OS of patients in the high‐risk group was lower, and the AUC value of ROC performed well (Figure 3E, F). Through calculation, the R square of our model is about 0.70, which indicates that the predictive ability of our prognostic risk scoring model is acceptable.

### 3.5 Comparison of predictive performance of prognostic models

**FIGURE 4:**
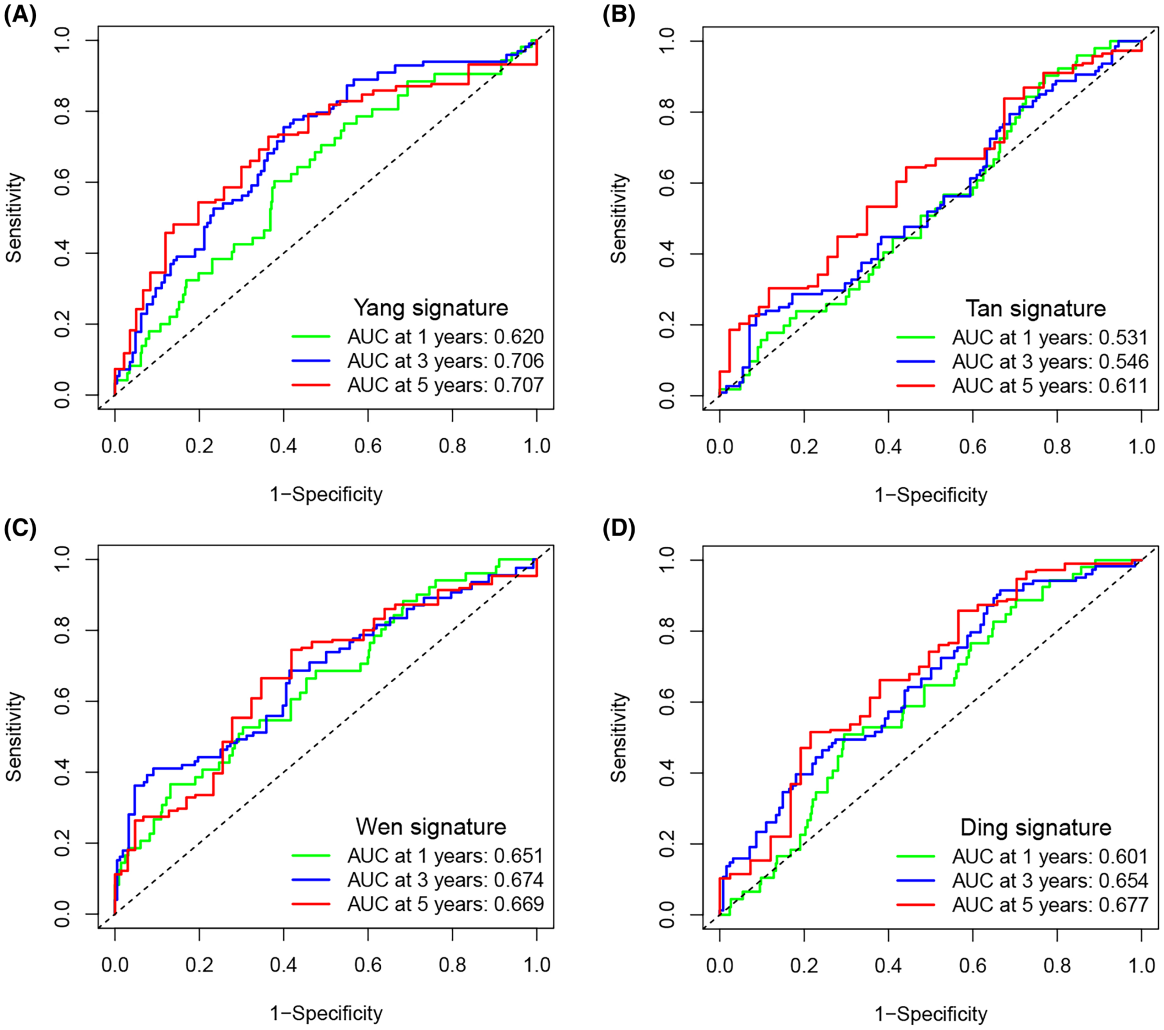
Predictive performance of different prognostic models. (A) ROC of model Yang signature. (B) ROC of model Tan signature. (C) ROC of model Wen signature. (D) ROC of model Ding signature.

In order to verify the superiority of the ER stress prognostic risk scoring model, the models in previous CRC studies were compared with our model through ROC. The first is a model for studying lipid metabolism, which is called Yang signature according to the name of the researcher.^15^ The second is a model for studying methylation, referred to as Tan signature.^16^ The third is the model for studying immune‐related genes, referred to as Wen signature.^17^ The fourth is a model for studying fatty acid metabolism, referred to as and Ding signature.^18^ According to the genes in the model, we used the R package for ROC analysis. The results showed that the AUC values of the ERS prognostic risk scoring model we constructed in 1, 3, 5 years were higher than those of the other four models (Figure 4A–D). This means that our risk scoring model has better prediction performance.

### 3.6 Association of clinicopathological features with risk scores

**FIGURE 5:**
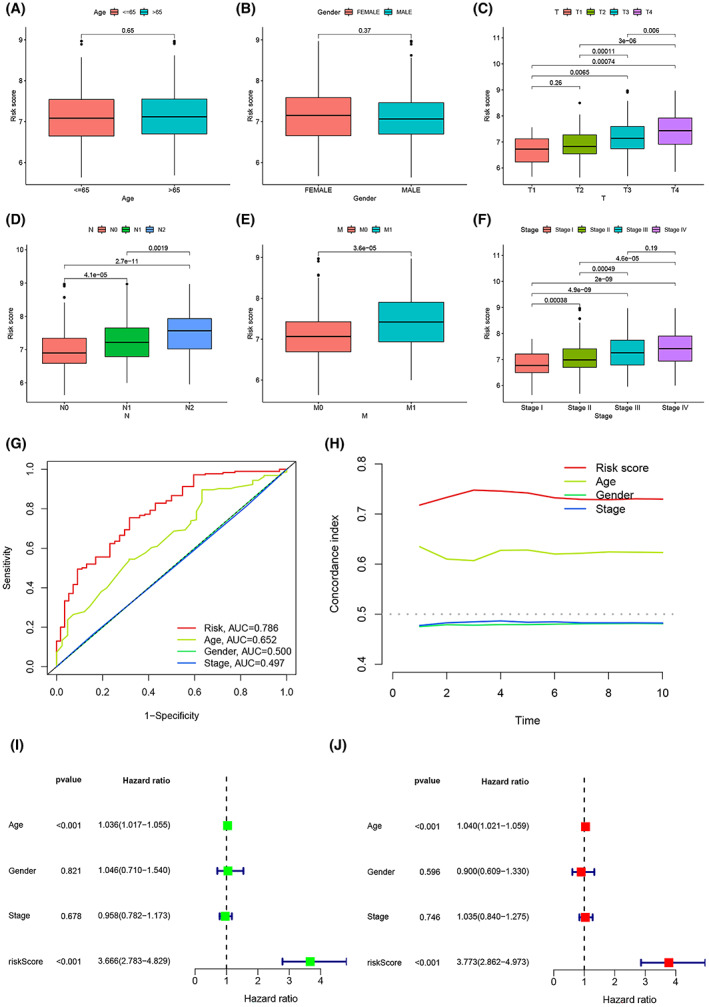
The relationship between risk scoring model and clinical features. (A–F) The relationship of risk score and clinicopathological features, including age (A), gender (B), tumor invasion (C), lymphoid metastasis (D), distal metastasis (E), and pathological stage (F). (G) Comparison of the predictive power of the risk model with that of other clinicopathological features. (H) C‐index of risk scoring model. (I, J) Forest plots for univariate and multivariate Cox regression analysis of risk scoring model.

Since different clinicopathological features have different effects on disease prognosis, the distribution of clinicopathological features in the risk score model was also explored. The results suggest that risk scores are most strongly associated with tumor progression. Although risk scores did not differ significantly with age or gender (Figure 5A, B), they did increase with higher TNM stage as well as higher pathological stage (Figure 5C–F). Next, the predictive ability of risk score, age, gender, and pathological stage on prognosis was compared, with the risk score having the highest AUC (0.786), indicating that the risk score showed the best predictive ability (Figure 5G). The C‐index showed that the risk score performed better than other clinical features and the accuracy of the prediction results was higher (Figure 5H). Figure 5I,J indicate that age and risk score were shown to be independent predictors of OS (*p* < 0.001).

### 3.7 Construction of a nomogram for predicting survival

**FIGURE 6:**
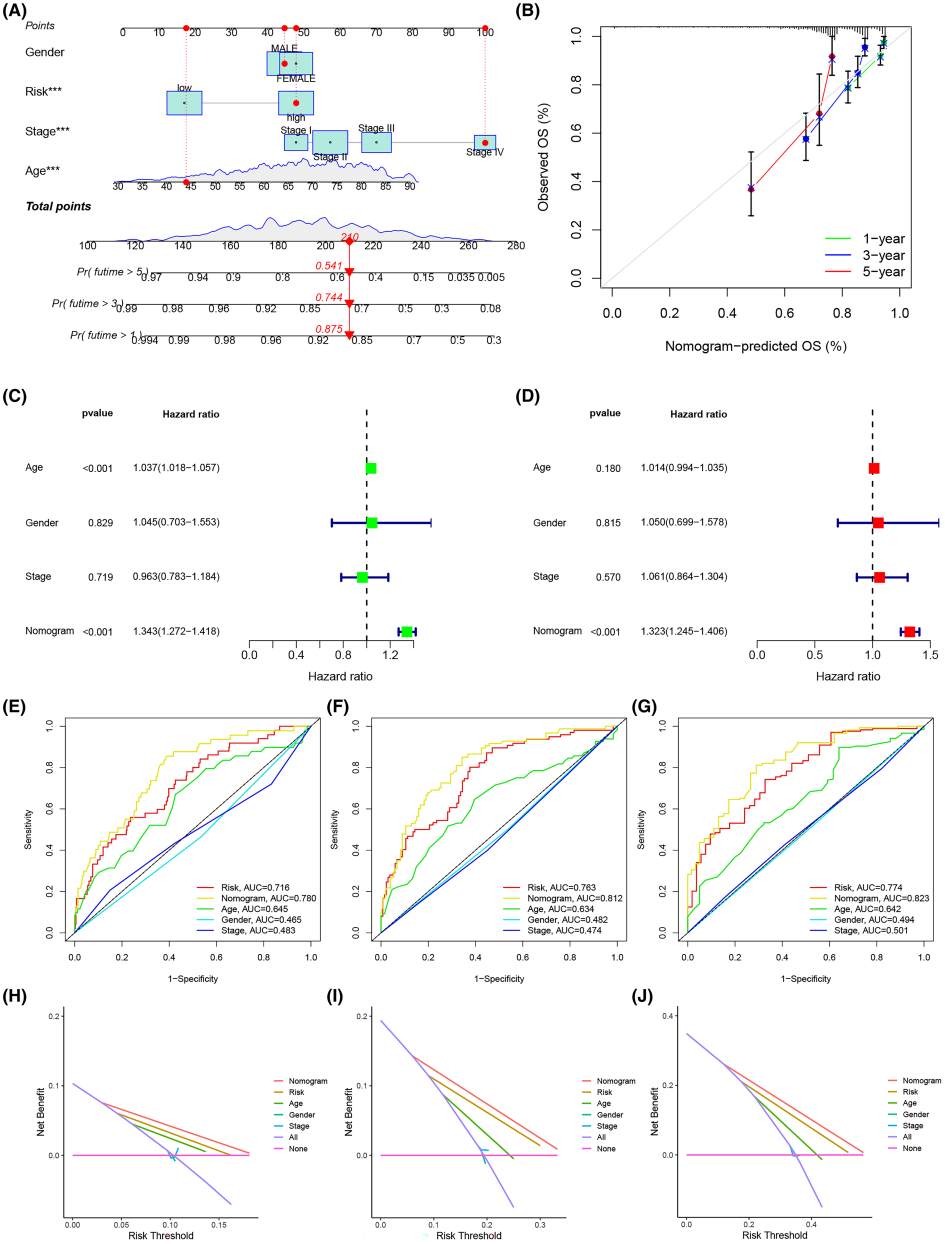
A nomogram to predict CRC prognosis was created by integrating ERS risk scores and clinicopathological features from the training set. (A) Nomogram for predicting the OS of patients in the training set. (B) Calibration chart for the nomogram. (C, D) Forest plots for univariate and multivariate Cox regression analysis of the training set. (E‐G) ROC curves for ERS risk scores and clinicopathological characteristics at 1‐ (E), 3‐ (F), and 5‐ (G) year. (H–J) DCA of the nomogram for 1‐ (H), 3‐ (I), and 5‐ (J) year OS in the training set.

A nomogram predicting OS in CRC was constructed by combining gender, risk score, pathological stage, and age from the training set (Figure 6A). The calibration curves for 1, 3 and 5 years demonstrate the accuracy of the nomogram (Figure 6B). Cox regression analysis eventually revealed that only nomogram was an independent predictor (*p* < 0.05) (Figure 6C,D). Figure 6E–G show the AUC for risk score, nomogram, age, gender, and pathological stage at 1, 3, and 5 years, with the nomogram having the highest AUC of 0.780, 0.812, and 0.823, respectively. Figure 6H–J shows the DCA analysis containing various clinicopathological features at 1, 3 and 5 years. The tree plots visually show that the nomogram for the prognostic risk score model performs best in the DCA curves at 1, 3, and 5 years.

### 3.8 Response to chemotherapy and changes in expression of related genes

**FIGURE 7:**
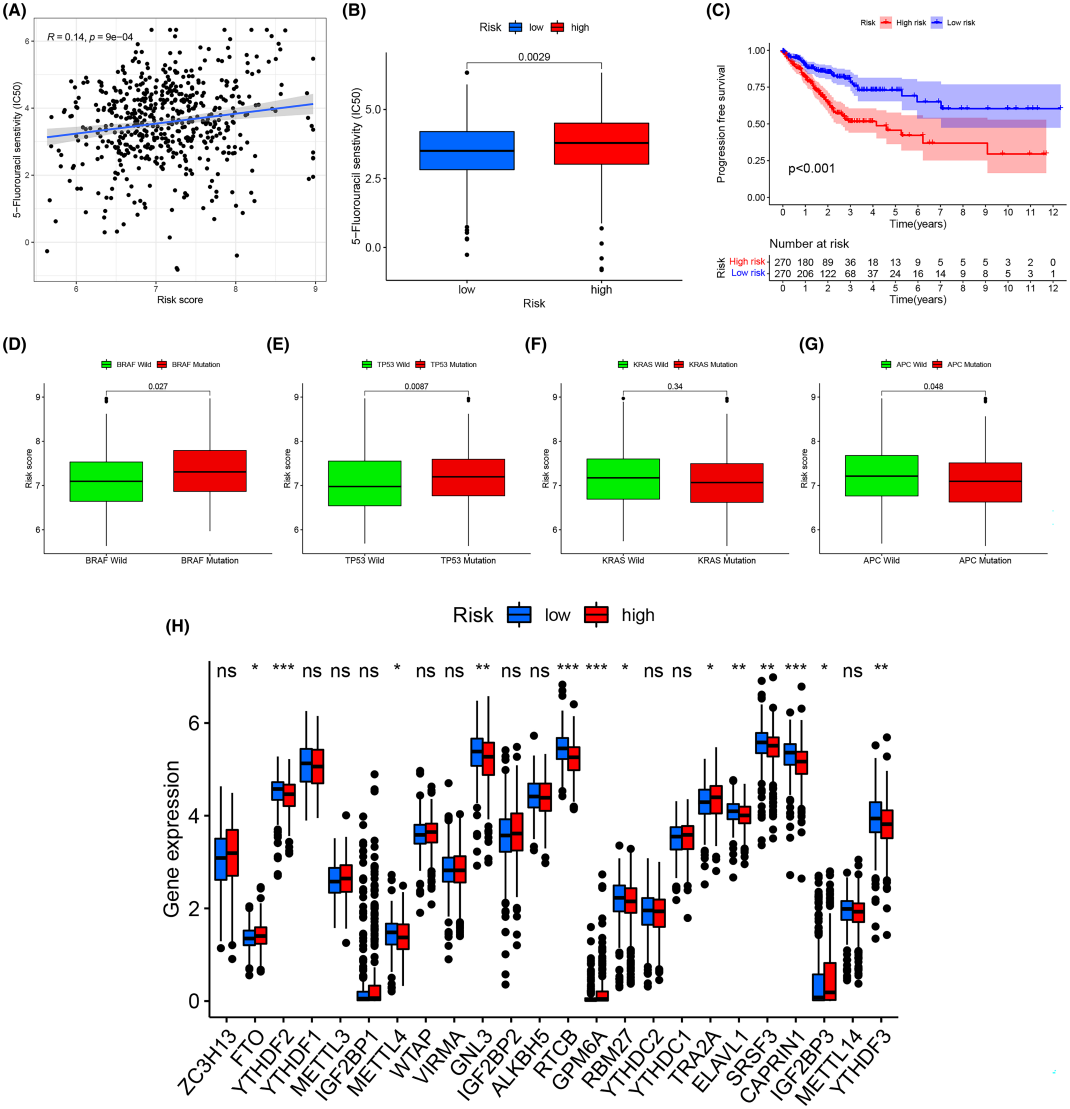
The role of the ERS prognostic risk score model in chemotherapy. (A) Differences in the efficacy of 5‐FU between patients in the high‐risk and low‐risk groups. (B) Correlation between patients' risk scores and the estimated IC50 for 5‐FU. (C) Comparison of PFS between the high‐risk and low‐risk groups in the training set. (D‐G) Differences in ERS risk score among different of molecule subtypes, including BRAF mutation (D), TP53 mutation (E), KRAS mutation (F), APC mutation (G). (H) Expression of m6A‐related genes in the high‐risk and low‐risk groups.

Since patients with CRC are routinely treated with chemotherapy after surgery, the response of patients in the model to chemotherapeutic agents was explored. The “pRRophetic” in the R software was used to analyze the association between risk scores and chemotherapy treatment outcomes. Samples in the low‐risk group were better treated with 5‐FU, and the IC50 increased with higher risk scores (Figure 7A,B). Progression‐free survival (PFS) also showed significant differences between subgroups in the training set, suggesting that risk scores can stratify chemotherapy‐resistant patients(*p* < 0.001) (Figure 7C). Mutant BRAF and TP53 had a high‐risk score, as did wild‐type APC (Figure 7D–G). In addition, most of the genes associated with m6A differed significantly in the ERS prognostic risk score model such as YTHDF2, YTHDF3, FTO, METTL4, RTCB, GPM6A, SRSF3, and CAPRIN1, which could provide help in finding therapeutic targets for chemotherapy resistance (Figure 7H).

### 3.9 Immune‐related features in the ERS prognostic model

**FIGURE 8:**
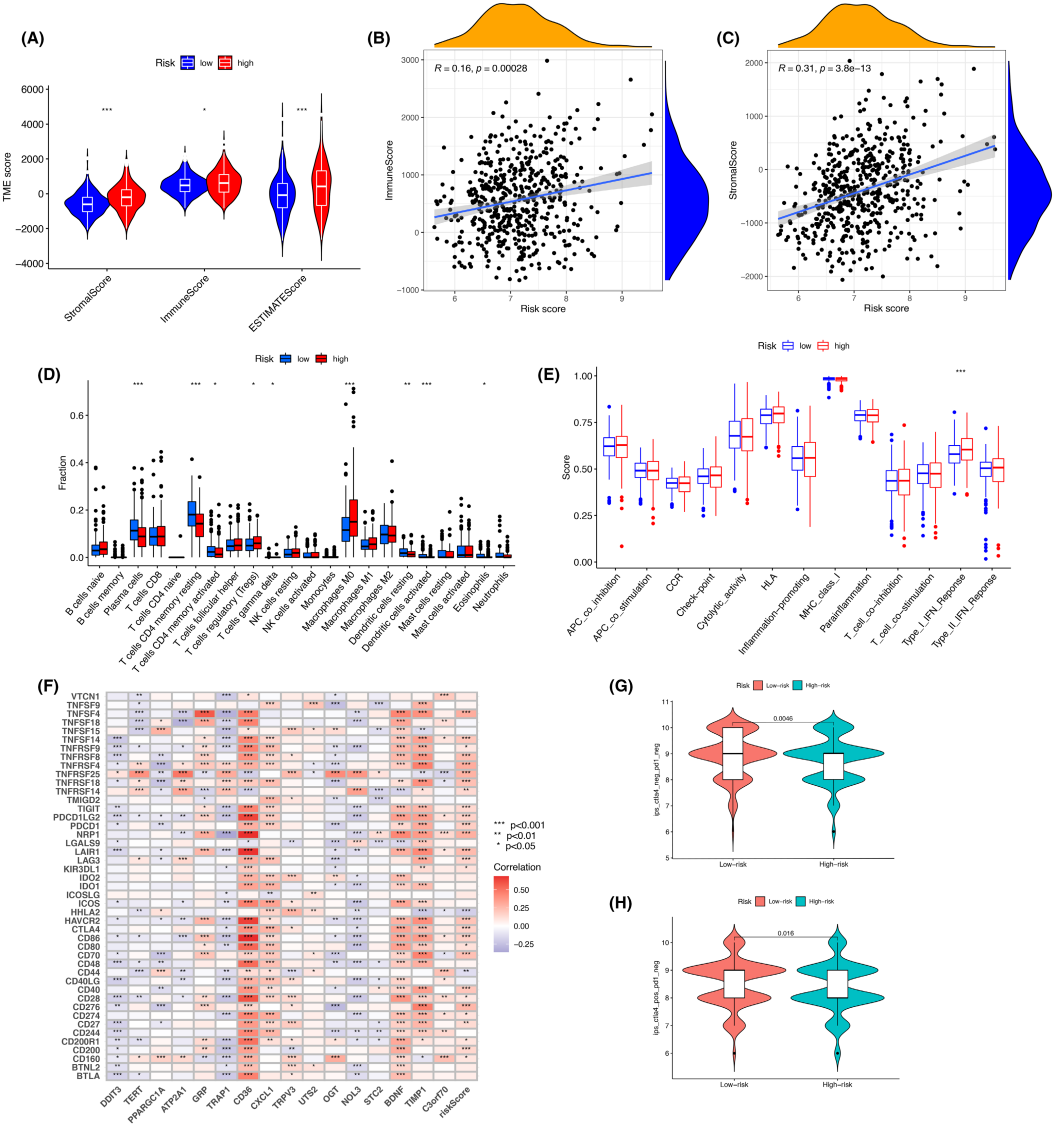
Immune‐related features in the ERS prognostic risk score model. (A) ESTIMATE scores, stromal scores, and immune scores for the different groups were assessed by ESTIMATE. (B) Correlation between immune score and risk score. (C) Correlation between stromal score and risk score. (D) Comparison of immune cell subtypes in the high‐risk and low‐risk groups. (E) Analysis of differences in immunomodulation‐related functions between the high‐risk and low‐risk groups. (F) Association of risk scores and prognosis‐related genes with checkpoints. (G) Samples without anti‐PD1 and anti‐CTLA4 immunotherapy in CRC. (H) Samples from CRC treated with anti‐CTLA4 immunotherapy only.

ESTIMATE score (*p* < 0.001), immune score (*p* < 0.05) and stromal score (*p* < 0.001) were significantly different between the two groups, indicating that ERS profoundly affects immune and stromal cell infiltration (Figure 8A). Figure 8B, C show the correlation of immune score and stromal score with risk score, respectively. Next, risk scores for invasive immune cells were studied using different software (FigureS3A). In addition, we found significantly more abundant immunosuppressive cell infiltration and high levels of regulatory T cells (Tregs) in the high‐risk group, which was consistent with a survival disadvantage in the high‐risk group, while T cell CD4 memory resting (*p* < 0.001), dendritic cells resting (*p* < 0.01), dendritic cells activated (*p* < 0.001), and plasma cells (*p* < 0.001) of low level of infiltration (Figure 8D). In previous reports, activation of type I interferon (IFN‐I) signaling could benefit tumor patients in immunotherapy.^19^, ^20^, ^21^ In this study, IFN‐I response was enhanced in the high‐risk group, implying that immunosuppressed patients can improve their prognosis with immunotherapy (Figure 8E). To predict the response of CRC patients to immunotherapy, the correlation between immune checkpoints and ERS was further investigated (Figure 8F). Our results showed that ERS risk score was positively correlated with most immune checkpoints, such as PDCD1 (PD‐1), CD274 (PD‐L1), CTLA‐4, LAG3, TIGIT, and HAVCR2 (TIM‐3). In addition, we found that PD‐1, TIGIT, TIM‐3, and CTLA‐4 expression was higher in the high‐risk group (Figure S3B). The ability of the ERS risk model to predict CRC prognosis was explored through an immunotherapy cohort (Table S3). The results showed that patients in the high‐risk group had a significantly poorer response to immunotherapy in the absence of any immunotherapy, and this result was maintained when immunotherapy only against CTLA4 was administered (Figure 8G, H). In addition, TIDE scores for the training set were obtained (Table S4) and analyzed between the ERS risk groups (Figure S3C). As TIDE prediction scores increased, prognostic outcomes decreased, indicating again that the low‐risk group exhibited better prognoses. Next, analysis of immunosuppressive gene expression in different subgroups of the model revealed that most immunosuppressive genes were expressed at increased levels in the high‐risk group (Figure S3D), which may account for the poor immunotherapy effect in this group of patients.

### 3.10 Functional enrichment analysis and PPI network of DEGs in the model

**FIGURE 9:**
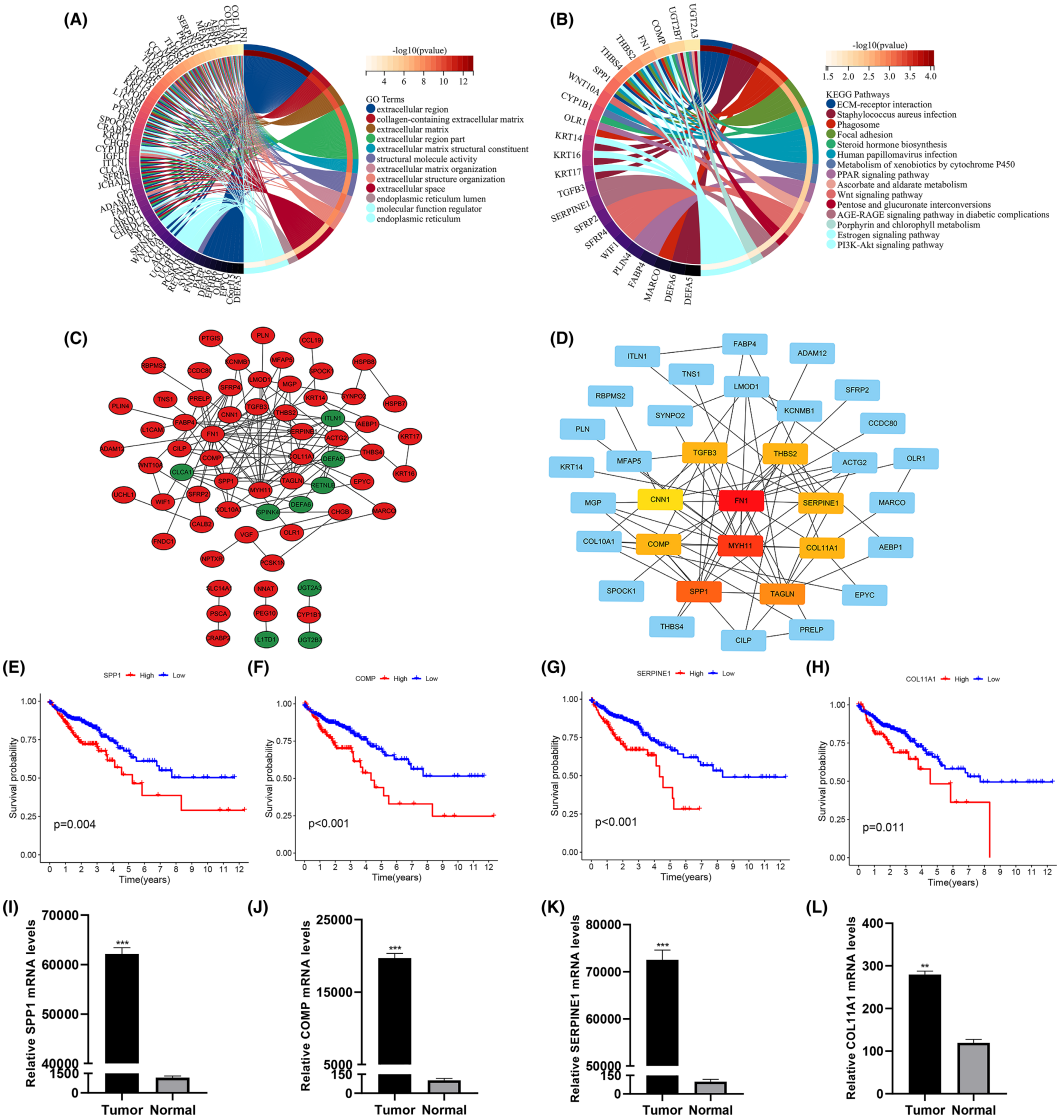
Visualization of the PPI network. (A, B) Circle diagram for GO and KEGG analysis. (C) PPI network processed by Cytoscape. Red represents DEGs expressing up‐regulation. blue represents DEGs expressing down‐regulation. (D) Top 10 hub genes selected by cytoHubba. (E–H) Survival analysis of SPP1(E), COMP(F), SERPINE1(G) and COL11A1(H). (I–L) qRT‐PCR analysis to validate the expression of SPP1(I), COMP(J), SERPINE1(K), and COL11A1(L) in normal and tumor tissues.

To better understand the role of the genes, functional enrichment analysis was performed on DEGs from both risk groups. GO results showed that these genes are involved in pathways including extracellular regions, extracellular region fractions, structural molecular activity, molecular functional regulators, ER, and extracellular structural organization (Figure 9A). KEGG analysis showed significant enrichment for such terms as human papillomavirus infection, ECM‐receptor interaction, phagosome, PI3K‐Akt signaling pathway, WNT signaling pathway, PPAR signaling pathway, and estrogen signaling pathway (Figure 9B). The online site STRING was used to study the protein interactions of DEGs in the risk group, and PPI networks were duly generated (Figure S4A). Visualization of the PPI data was done in Cytoscape software, with elevated and decreased gene expression marked in red and green, respectively (Figure 9C). Then Cytoscape's plugin cytoHubba was used to filter the hub genes in the DEGs, and the top 10 were ranked as shown in Figure 9D. The differences in hub genes were then compared between normal and tumor tissue. The results revealed that SPP1, COMP, THBS2, SERPINE1, and COL11A1 were highly expressed in tumor tissues, while MYH11, TAGLN, and CNN1 were lowly expressed (Figure S4B–I). Prognostic value analysis showed that of the eight hub genes mentioned above with statistically significant differences, only the expression of SPP1, TAGLN, COMP, SERPINE1, COL11A1, and CNN1 mRNAs affected the prognosis (Figures 9E–H; Figure S4J–M), with levels of SPP1, COMP, SERPINE1, and COL11A1 being, more specifically, associated with poor prognosis. To verify the differential expression of SPP1, COMP, SERPINE1, and COL11A1 between normal and CRC tissues, q‐PCR was performed on normal and tumor biopsies from 11 CRC patients (Figure 9I–L).

## 4 DISCUSSION

CRC is a malignant disease of the gastrointestinal tract with high morbidity and mortality. Despite advances in surgery and chemoradiotherapy, the prognosis of CRC remains unsatisfactory. Therefore, exploring new prognostic indicators and treatment methods are critical.

Recently, ERS has become an increasingly attractive research field across various cancer types. During ERS, a series of intracellular stimuli activate UPR and integrate signal transduction pathways through three different receptors, IRE1α, PERK, and ATF6, to restore the balance of ER. When ERS leads to UPR, it either promotes cell survival by increasing the level of protein folding, or leads to apoptosis.^22^ Numerous studies have pointed out that ERS is not only involved in the development of multiple tumors but can also affect chemoresistance, immune function and invasive metastasis of tumor cells.^23^, ^24^, ^25^ In CRC, when ERS, IRE1α is activated to produce XBP1s by cutting mRNA, while XBP1s acts as a transcription factor binding to IRE1α promoter to transcribe IRE1 α to form an IRE1α‐XBP1s axis to induce cancer cell proliferation.^26^ In the process of ERS‐induced epithelial‐mesenchymal transformation (EMT), GRP78/PERK signaling pathway is activated, which promotes nuclear translocation of ATF4 and increases the transcriptional activity of interleukin‐like met inducers, which accelerates tumor progression.^27^ In pancreatic cancer, activation of ERS induces the epithelial‐mesenchymal transition, which exacerbates tumor cell invasion and metastasis and contributes to accelerated tumor progression.^28^ Importantly, ERS has also been shown to induce activation of the eIF2α/ATF4 axis, and increased eIF2α phosphorylation leads to increased chemoresistance in CRC.^29^ In addition, ERS can also influence therapeutic efficacy–including that of immunotherapy–by reshaping the tumor microenvironment.^30^ However, the combined role of ERSRGs is not fully clear, and their impact on CRC necessitates additional investigation.

In this study, a new risk score model was developed to assess the impact of ERS on the prognosis of CRC. PCA analysis showed that the prognostic risk score model was effective in stratifying patients with CRC. Compared with other prognostic studies of CRC, we have constructed a risk curve and risk histogram that can more intuitively show that death increases with the increase of risk score. In terms of clinicopathological factors, although our model performs poorly in terms of age and sex compared with other models, our model has great advantages in distinguishing T, N, M stages and pathological stages. Kaplan–Meier curves show significantly shorter survival times for patients in the high‐risk group. The validation sets (GSE40967 and GSE17538) validate the results of the training set and shows that the predictive power of our risk scoring model is reliable. However, in the validation set GSE40967, although our model performed well in predicting OS, it performed poorly in predicting 1‐, 3‐, and 5‐year survival (AUC = 0.61). In the validation set GSE17538, the 1‐, 3‐ and 5‐year survivals performed well (AUC = 0.77). This may be due to other reasons such as geography, race, data collection methods and sample testing methods. Therefore, in order to further verify the prediction performance of the model, we will further verify the data collected by ourselves and the data set GSE32323. Compared with the models of other studies, the prognostic model we constructed has the best predictive performance. In addition, nomograms combining risk scores and clinicopathological features were used to explore the potential of the model. The results show that the prediction ability of the nomograms constructed by us has been further improved. The performance of our established nomograms was confirmed by calibration plots and DCA analysis. Compared with other studies, the DCA decision curve we introduced can help clinicians.

We also investigated the effect of ERS on CRC chemotherapy. Not only was the high‐risk group more resistant to the chemotherapeutic agent 5‐FU, but the risk score was positively correlated with resistance to 5‐FU. Meanwhile, patients with high‐risk scores had lower PFS. In conclusion, our risk score model can provide prediction of 5‐FU efficacy for patients. Gene mutations have been reported to cause poor prognosis due to chemotherapy resistance in tumor patients, and this study also analyzed common gene mutations in CRC.^31^, ^32^, ^33^ Our study found that mutated BRAF and TP53 have higher risk scores, which means that patients have a worse prognosis. Since mutations in BRAF and TP53 genes usually lead to poor outcomes of chemotherapeutic drugs or targeted drugs, our risk model can also provide predictions for patients' drug therapy.^34^ Recent studies have identified m6A‐related genes involved in the resistance to chemotherapies, immunotherapies, and other targeted therapies in some tumors.^35^, ^36^ Studies have also reported that m6A‐related genes are involved in the development of CRC.^37^, ^38^, ^39^ Therefore, this study also explored the relationship between risk models and m6A‐associated genes. The results showed that m6A‐related genes showed significant differences in the models, however, the specific mechanisms of action of these genes in CRC need to be further investigated.

ERS affects a variety of immune processes, including the production of pro‐inflammatory cytokines, regulation of immune cells, antigen presentation, monitoring of cellular status, and maintenance of immune tolerance.^40^ ERS has also been shown to influence tumor immunity. In breast cancer, ERS promotes miR‐27a‐3p expression, which then upregulates PD‐L1 through the MAGI2/PTEN/PI3K axis, ultimately contributing to immune evasion by the tumor.^14^ In this study, immune microenvironment analysis showed that patients with high‐risk scores had high ESTIMATE, immune and stromal scores. This may be one of the reasons for the high mortality rate of patients in the high‐risk group. As major components of the tumor microenvironment, immune cells are also capable of being affected by ERS, which can alter tumor cell progression.^30^ Based on differential immune cell analysis, we found a higher enrichment of Tregs and immunosuppression‐associated macrophages in the high‐risk group. Tregs are immunosuppressive cells that can directly kill effector T cells as well as block their cellular messages via secretion of inhibitory cytokines.^41^ The high infiltration of Treg cells leads to increased mortality in CRC patients.^42^, ^43^ Immune checkpoints are potential targets for cancer treatment, and inhibitors blocking key checkpoint molecules have shown impressive anti‐cancer effects. In this study, 45 immune checkpoint‐associated genes were analyzed, and the risk score was able to assess the impact of immune checkpoints on immunotherapy. Analysis of TCIA and TIDE scores showed a poorer response in the high‐risk group, possibly due to the combination of multiple ERS pathways altering the cellular immune status and thus leading to immune escape.^10^, ^44^ Moreover, some specific immunosuppressive genes were elevated in the high‐risk group, which may also contribute to the poorer outcome of immunotherapy in this group of patients. Taken together, targeting ERS in tumor cells may help to enhance the efficacy of current forms of tumor immunotherapy.

In order to further explore the role of ERSRGs in CRC, we analyzed the DEGs of two subgroups with significant differences. SPP1, COMP, SERPINE1, and COL11A1 were found to be essential. SPP1 is a secreted glycoprotein that affects the adhesion, proliferation, differentiation, migration, and survival of a variety of cells.^45^, ^46^ In CRC, increased expression of SPP1 affects the growth, invasion, and metastasis of CRC cells.^47^ COMP is a soluble pentameric glycoprotein that affects the stability of the extracellular matrix.^48^ COMP has also been shown to collaborate with the EMT pathway, leading to more aggressive tumor cells.^49^ SERPINE1, an activator of tissue fibrinogen and inhibitor of adenosine uridylate phosphate, was shown to be involved in ovarian cancer chemoresistance via the EMT pathway.^50^ A study has shown that SERPINE1 expression is enhanced in CRC and is associated with tumor aggressiveness, but the exact mechanism of this effect remains to be investigated.^51^ COL11A1 is an isoform of fibrillar collagen that is involved in the proliferation, migration and apoptosis of cancer cells.^45^, ^52^ In CRC, high expression of COL11A1 was not only positively correlated with the tumor stage but also with the malignant behavior of CRC, and miR‐339‐5p could down‐regulate COL11A1 expression and inhibit colorectal progression.^53^ We have verified the mRNA levels of these genes in CRC specimens by qRT‐PCR; however, further basic studies are needed to elucidate their functions in CRC. In CRC, the discovery of ERS‐related prognostic key genes has provided new clues to explore the molecular pathways of ERS and new ideas for the study of targeted therapies in CRC.

There are some limitations to this study. First, our data are from public datasets with incomplete relevant clinical information, and therefore more multicentre clinical specimens would be beneficial in understanding the role of ERS in CRC. Second, it is not clear why immune checkpoint‐related gene expression is high in the high‐risk group, but the response to immunotherapy against immune checkpoints is poor; more in‐depth studies will be required to reveal the reasons for this. Finally, further basic experimentation based upon this work would be critical in determining the specific mechanism of action of ERS in CRC.

## 5 CONCLUSION

This study provides a new and accurate protocol for predicting the prognosis of CRC patients. In view of the considerable prognostic ability of our model and its accurate prediction of treatment outcomes, our risk score model can be used as a new tool for patients to choose treatment. Given the importance of ERS in CRC, a more in‐depth study of its specific mechanism of action is warranted.

## AUTHOR CONTRIBUTIONS

**Jingbo Geng:** Data curation (equal); software (equal); visualization (equal); writing – original draft (equal). **Yunkai Guo:** Formal analysis (equal); software (equal). **Mingjie Xie:** Methodology (equal); supervision (supporting); validation (supporting). **Zhipeng Li:** Investigation (equal); methodology (equal); software (equal); supervision (equal); validation (equal). **Peng Wang:** Methodology (supporting); software (supporting). **Donghui Zhu:** Data curation (supporting); methodology (supporting); software (supporting); visualization (supporting). **Jing Li:** Funding acquisition (equal); project administration (equal); writing – original draft (equal); writing – review and editing (equal). **Xiaopeng Cui:** Conceptualization (lead); resources (lead).

## CONFLICT OF INTEREST STATEMENT

The authors declare that we do not have any possible conflict of interest.

## FUNDING STATEMENT

Funding for this study was obtained from the Nantong Science and Technology Foundation grant (MS22019008, JC22022009, JC22022027), the Project of Health Committee of Nantong (QA2020018, MB2021046), and by Nantong University Pre‐Research Project (17ZY36).

## ETHICS STATEMENT

This study was approved by the Ethics Committee of Nantong University Hospital (project number: 2022‐L090). All patients signed an informed consent form.

## Supporting information


Figure S1.

Figure S2.

Figure S3.

Figure S4.

Table S1.

Supplementary Table S2.

Supplementary Table S3.

Supplementary Table S4.

## Data Availability

The validation set data was deposited in NCBIGEO (https://www.ncbi.nlm.nih.gov. Accessed 26 May 2022). The training set data was downloaded from the TCGA official website (http://gdc.cancer.gov. Accessed 26 May 2022). Endoplasmic reticulum stress gene set from GeneCards database (https://www.genecards.org/. Accessed 26 May 2022). TCIA pair column data from online site (http://tcia.at/. Accessed 23 July 2022). TIDE data from a web page (http://tide.dfci.harvard.edu/. Accessed 23 July 2022).
