# Supplementary figures and images for: Characteristics of endoplasmic reticulum stress in colorectal cancer for predicting prognosis and developing treatment options
Source: Cancer Med. 2023 Mar 31;12(10):12000–17. doi: 10.1002/cam4.5874 (PMC10242314; doi:10.1002/cam4.5874)

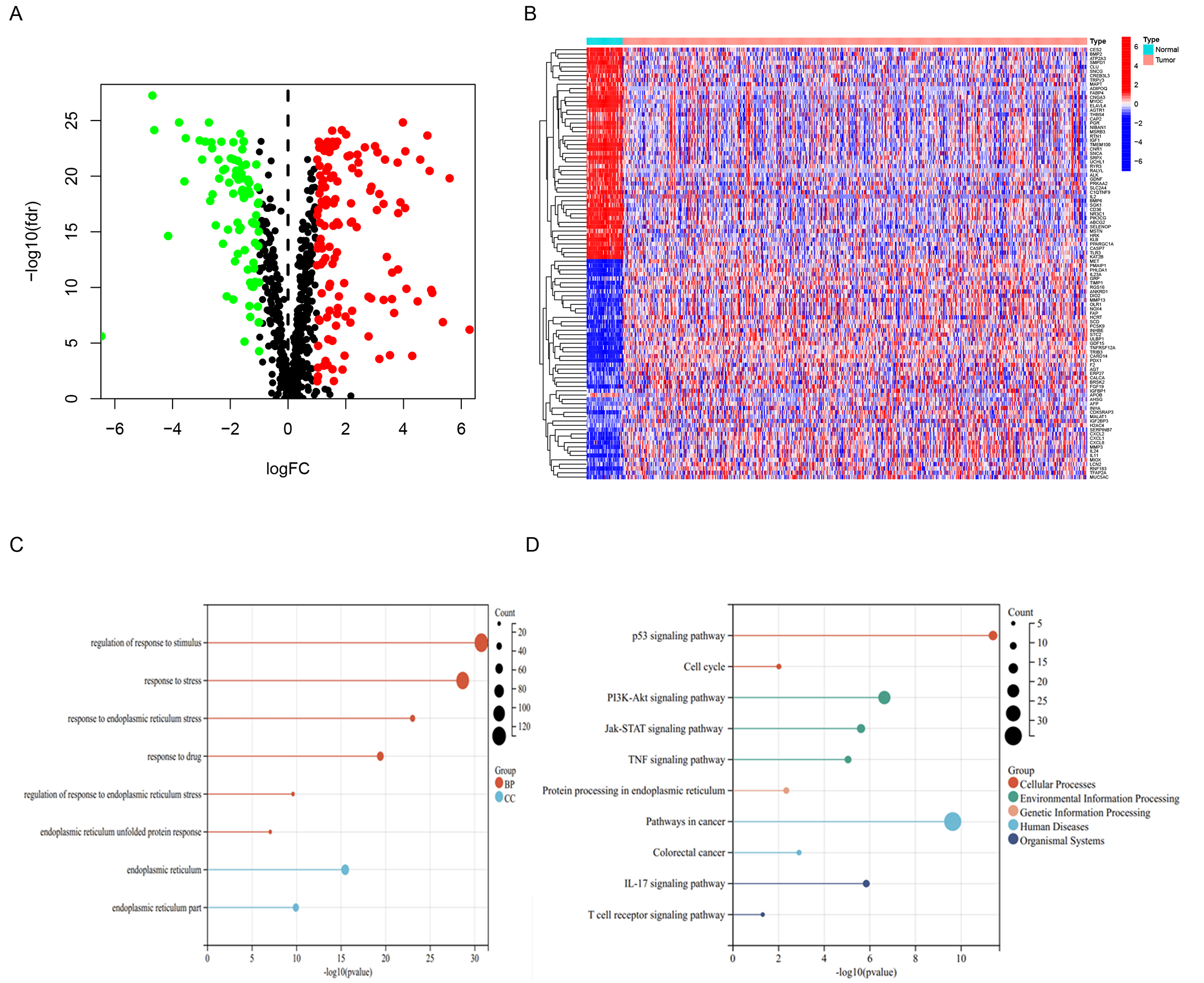

Supplement: Supplementary file 1 — Figure S1. [file CAM4-12-12000-s003.tif]

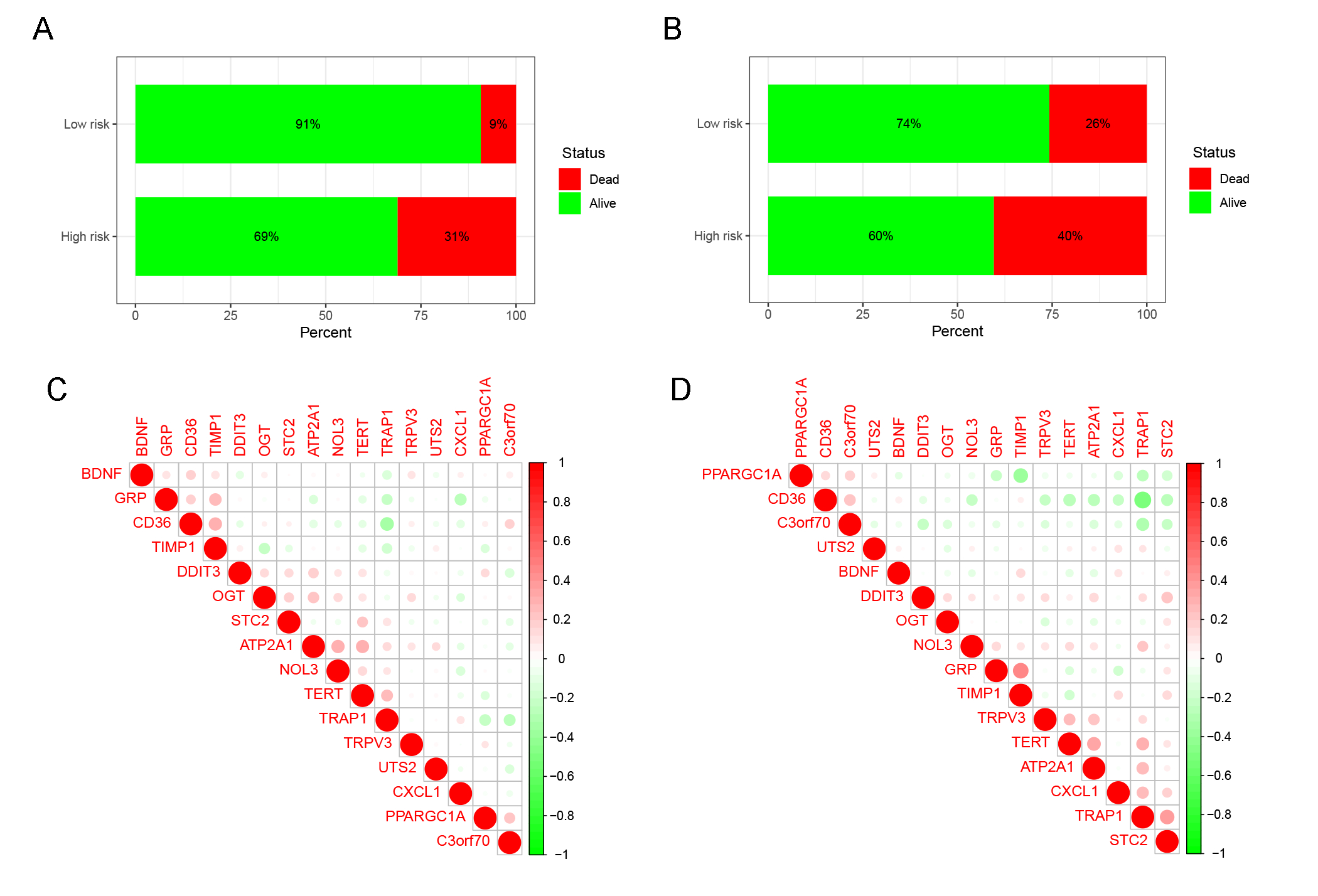

Supplement: Supplementary file 2 — Figure S2. [file CAM4-12-12000-s007.tiff]

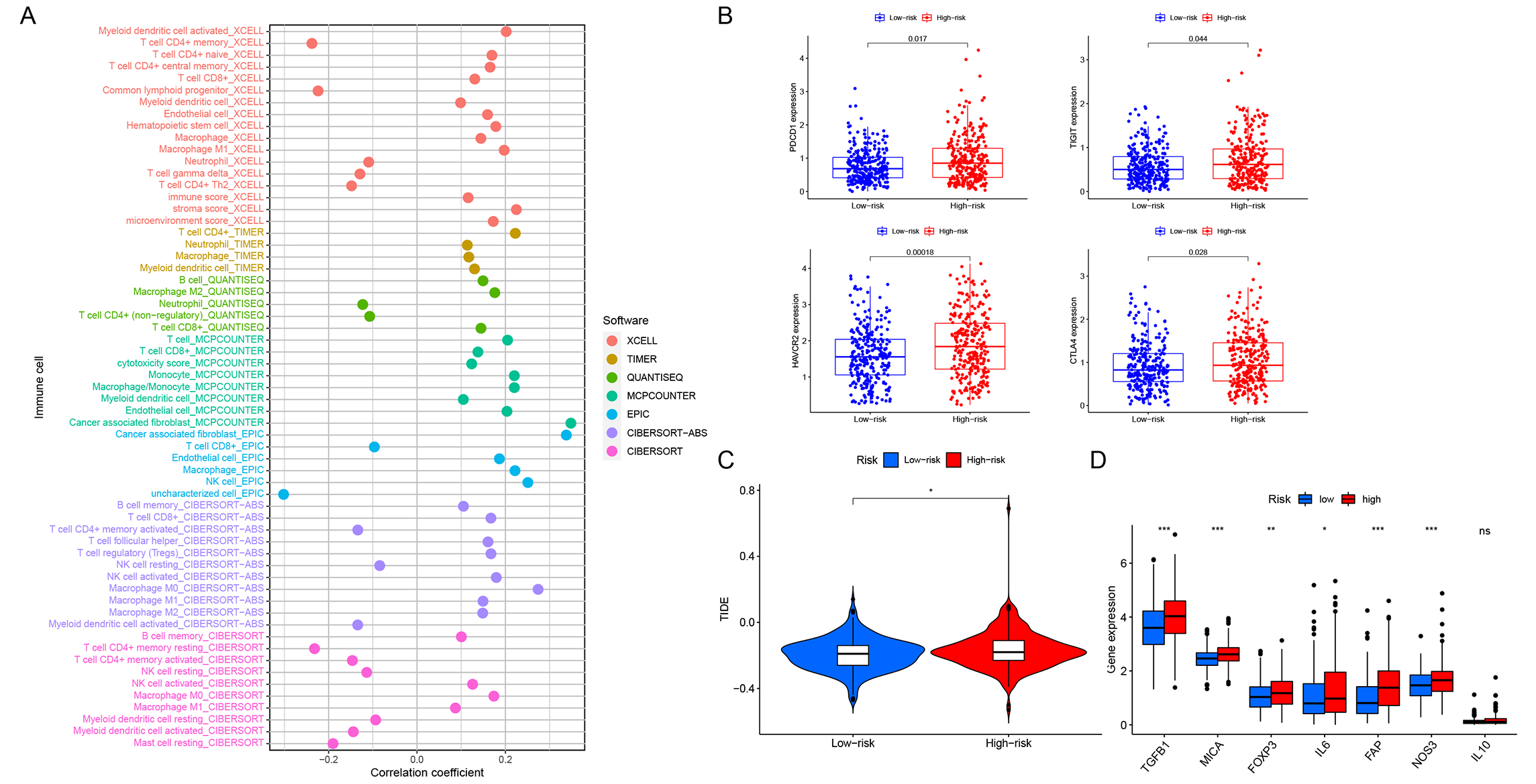

Supplement: Supplementary file 3 — Figure S3. [file CAM4-12-12000-s005.tif]

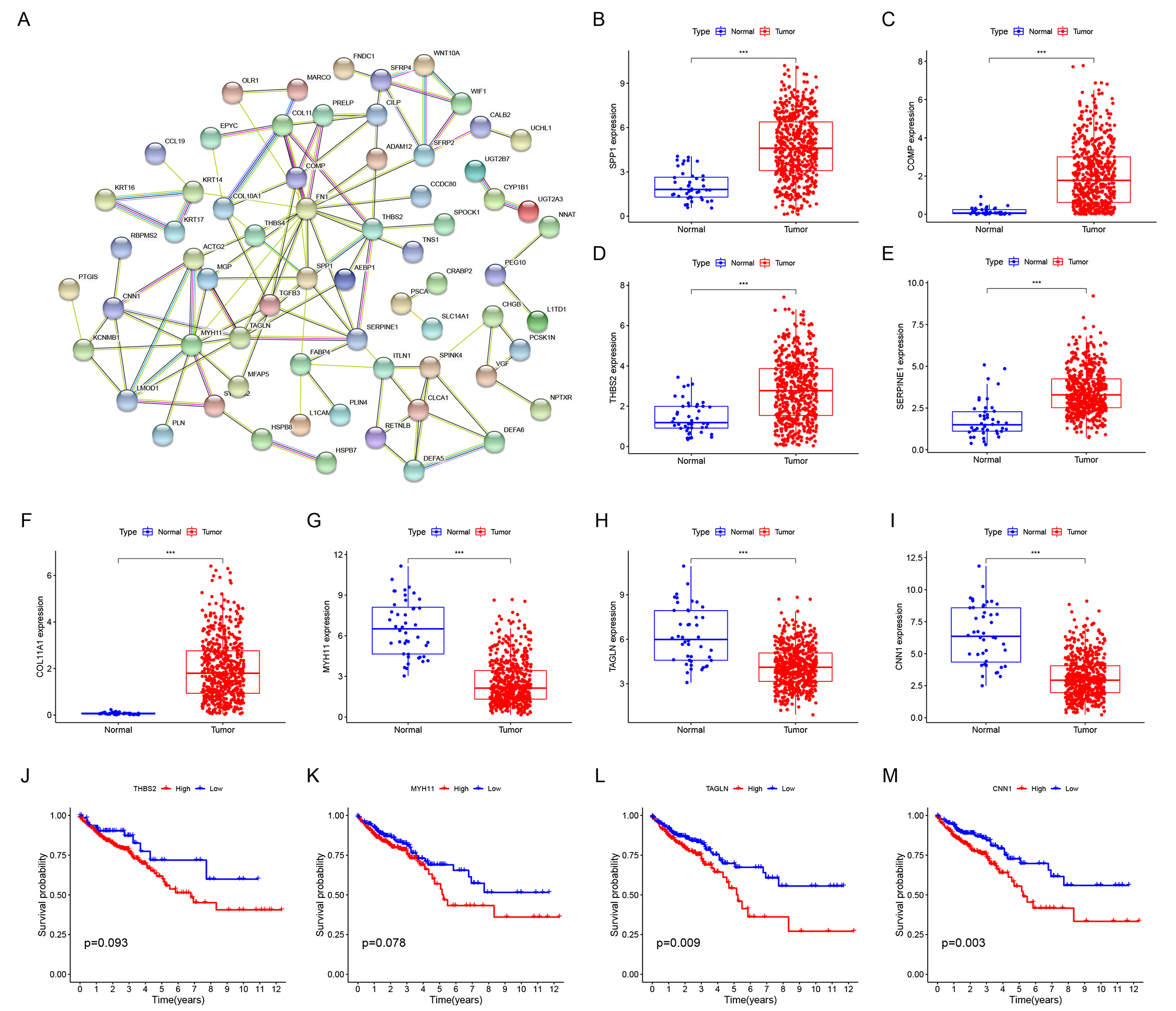

Supplement: Supplementary file 4 — Figure S4. [file CAM4-12-12000-s001.tif]
